# Baseline characteristics and outcome for aneurysmal versus non-aneurysmal subarachnoid hemorrhage: a prospective cohort study

**DOI:** 10.1007/s10143-021-01650-x

**Published:** 2021-10-04

**Authors:** Catharina Conzen, Miriam Weiss, Walid Albanna, Katharina Seyfried, Tobias P. Schmidt, Omid Nikoubashman, Christian Stoppe, Hans Clusmann, Gerrit A. Schubert

**Affiliations:** 1 Department of Neurosurgery, University Hospital Aachen, RWTH Aachen University, Pauwelsstrasse 30, 52074 Aachen, Germany; 2 Department of Neuroradiology, University Hospital Aachen, RWTH Aachen University, Aachen, Germany; 3 Department of Intensive Care and Intermediate Care, University Hospital Aachen, RWTH Aachen University, Aachen, Germany; 4 Department of Neurosurgery, Kantonsspital Aarau, Aarau, Switzerland

**Keywords:** Subarachnoid hemorrhage, Good grade, Perimesencephalic, Angiographically negative, Non-perimesencephalic, Delayed cerebral ischemia

## Abstract

This study aims to investigate the characteristics of patients with mild aneurysmal and non-aneurysmal perimesencephalic and non-perimesencephalic subarachnoid hemorrhage (aSAH, pmSAH, npmSAH) with emphasis on admission biomarkers, clinical course, and outcome. A prospective cohort of 115 patients with aSAH (Hunt and Hess 1–3) and of 35 patients without aneurysms (16 pmSAH and 19 npmSAH) admitted between January 2014 and January 2020 was included. Demographic data, blood samples on admission, complications (hydrocephalus, shunt dependency, delayed cerebral ischemia DCI, DCI-related infarction, and mortality), and outcome after 6 months were analyzed. Demographic data was comparable between all groups except for age (aSAH 55 [48–65] vs. npmSAH 60 [56–68] vs. pmSAH 52 [42–60], *p* = 0.032) and loss of consciousness (33% vs. 0% vs. 0%, *p* = 0.0004). Admission biomarkers showed poorer renal function and highest glucose levels for npmSAH patients. Complication rate in npmSAH was high and comparable to that of aSAH patients (hydrocephalus, shunt dependency, DCI, DCI-related infarction, mortality), but nearly absent in patients with pmSAH. Favorable outcome after 6 months was seen in 92.9% of pmSAH, 83.3% of npmSAH, and 62.7% of aSAH (*p* = 0.0264). In this prospective cohort of SAH patients, npmSAH was associated with a complicated clinical course, comparable to that of patients with aSAH. In contrast, such complications were nearly absent in pmSAH patients, suggesting fundamental differences in the pathophysiology of patients with different types of non-aneurysmal hemorrhage. Our findings underline the importance for a precise terminology according the hemorrhage etiology as a basis for more vigilant management of npmSAH patients. NCT02142166, 05/20/2014, retrospectively registered.

**Supplementary Information:**

The online version contains supplementary material available at 10.1007/s10143-021-01650-x.

## Introduction

In up to 15% of patients with spontaneous subarachnoid hemorrhage (SAH), no aneurysm as a source of hemorrhage can be identified [20, 24]. The underlying pathophysiology of non-aneurysmal SAH remains poorly understood, but previous studies implicated aberrant venous drainage patterns in the basal cisterns and thereby a low-pressure hemorrhage [3, 21]. According to the blood distribution on the initial CT scan, non-aneurysmal hemorrhages can be divided into perimesencephalic SAH (pmSAH, blood within the interpeduncular, ambient, and chiasmatic cistern) and non-perimesencephalic SAH (npmSAH, diffuse distribution of blood) [13, 20, 23]. The clinical course and outcomes of patients with pmSAH and npmSAH are commonly reported as rather benign, in particular in comparison with aSAH [6, 10, 13]. However, the assumption that patients suffering from SAH without evidence of an aneurysm do not require intensive neurological monitoring during the early stage of admission is increasingly challenged [1, 12, 19], but robust prospective data are scarce. Further, differences in the pathogenesis, clinical management, and outcomes across these three entities, i.e., aSAH, pmSAH, and npmSAH, have been seldom investigated in a balanced, prospective cohort. This study aims to characterize differences in the clinical course and neurological outcome across different types of SAH.

## Materials and methods

### Study design

This study was approved by the local ethics committee of the University Hospital of Aachen (Ethik-Kommission Uniklinik RWTH Aachen, Germany, IORG0006299) with the approval number EK 062/14 and was registered (NCT02142166). Data from all patients with spontaneous SAH admitted to our institution between January 2014 and January /2020 and meeting the following inclusion criteria were prospectively recorded: (1) patient age greater than 18 years, (2) SAH verified by CT scan or lumbar puncture when imaging was inconclusive, (3) Hunt and Hess 1–3 on admission, and (4) diagnosis or exclusion of a bleeding source was performed by digital subtraction angiography (DSA) including a 3D rotational run. Patients with traumatic SAH, SAH from other, non-aneurysmal vascular pathologies, or with intracerebral or subdural hemorrhage were excluded.

Written informed consent was acquired from all patients or their respective legal representatives. For balanced comparison, we only included aSAH patients with milder clinical presentation (Hunt and Hess 1–3) as patients with non-aneurysmal SAH present in the majority of cases with a good/mildly impaired neurological status (Hunt and Hess 1–3). Patients without evidence of a cerebral aneurysm were further stratified into two groups according to blood distribution. Hemorrhages restricted to the interpeduncular cistern with or without extension to the ambient, chiasmatic, and horizontal part of the Sylvian cistern were classified as pmSAH. Hemorrhage patterns with extension to the interhemispheric cisterns, convexity or focus in a different location than the interpeduncular cistern and with extension to the vertical portion of the Sylvian cistern were classified as npmSAH [13, 20, 23].

### SAH management algorithm

If an aneurysmal bleeding source was identified, it was secured within 48 h by either clipping or coiling following an interdisciplinary evaluation. All patients were closely monitored on a designated neurointensive care unit. Treatment of patients was conducted according to our local institutional standard operating procedure and has been described in detail elsewhere [27]. Acute hydrocephalus was addressed by insertion of an external ventricular drain. In patients, where clinical examination was precluded due to secondary clinical deterioration, regional neuromonitoring probes for cerebral microdialysis and brain tissue oxygen were inserted.

All patients with angiogram-negative SAH including 3D rotational reconstruction underwent early MRI scanning (cranium and cervical spine) to exclude other intra- or extraparenchymal sources of bleeding (i.e., cavernoma, fistula). In cases of a non-perimesencephalic blood distribution, an additional DSA was performed after 1 to 2 weeks to exclude an obscured vascular pathology.

### Data collection

All demographic and clinical data were collected prospectively. Blood samples and blood gas analysis within 24 h of admission were recorded, including leucocytes, electrolytes (sodium, potassium), renal panels (creatinine, glomerular filtration rate), glucose metabolism (glucose, lactate, glucose-potassium ratio), and C-reactive protein for inflammation.

### Radiological, metabolic, and clinical definitions

Primary outcome was defined as neurological outcome assessed after 6 months using the modified Rankin scale (mRS) during regular follow-ups or a structured telephone interview by a blinded investigator. The mRS scale was dichotomized into favorable (mRS 0–2) and unfavorable (mRS 3–6) outcome. The secondary outcomes were as follows: acute hydrocephalus, need for permanent CSF diversion (i.e., VP shunt), length of stay, DCI, DCI-related infarction, other cerebral infarction (e.g., intervention related), and mortality.

Delayed cerebral ischemia (DCI) was diagnosed according to the definition of Vergouwen et al. [25]: new focal neurological deficit or decrease in Glasgow coma scale ≥ 2 for a duration ≥ 1 h or reversible after treatment and not ascribable to other reasons (e.g., infection, hydrocephalus). This definition was expanded by functional parameters in analgosedated patients, who were neurologically not assessable: characteristic hypoperfusion on CT perfusion, usually triggered by oxygenation crisis (p_ti_O_2_ < 10 mmHg), and metabolic derangement as determined by cerebral microdialysis (lactate/pyruvate ratio ≥ 40). DCI-related infarction was diagnosed either on MRI or CT scan during hospitalization.

### Statistical analysis

Quantitative parameter values are presented as median [1. quartile – 3. quartile]. Normal distribution was tested using the Shapiro–Wilk and Kolmogorov–Smirnov normality tests. Differences between two groups were analyzed using the two-sided Student’s *t*-test or Mann–Whitney *U* test. Categorical variables were analyzed in contingency tables using the chi-square test resp. Fisher’s exact test. Comparisons between three groups were calculated with ANOVA or the Kruskal Wallis test, followed by Tukey’s multiple comparison test or Dunn’s multiple comparison test as appropriate.

Results are reported by *p*-values, odds ratio (OR), and 95% confidence intervals (CIs) where appropriate. For all comparisons, alpha level for statistical significance was set to 0.05. All analyses were performed using Numbers®, Apple Inc., Cupertino, USA, and GraphPad Prism®, GraphPad Software, Inc., La Jolla, USA.

## Results

### Baseline demographics and serum analysis for all SAH types

**Fig. 1:**
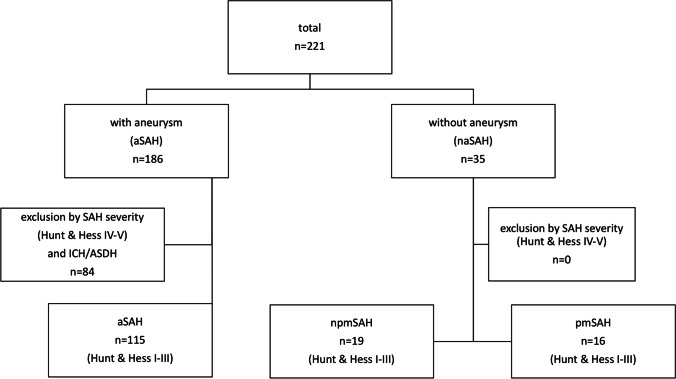
Flowchart of prospective cohort enrolment. In naSAH, no patient met the exclusion criteria Hunt and Hess 4 and 5, ASDH, or ICH. na, non-aneurysmal; npm, non-perimesencephalic; pm, perimesencephalic; ICH, intracerebral hemorrhage; aSDH, acute subdural hematoma

Out of the total of 221 SAH patients admitted to our hospital during the study period, 115 patients with good-grade aSAH (Hunt and Hess 1–3) were included (Fig. 1). These patients were compared with 35 SAH patients where no bleeding source could be identified (pmSAH: *n* = 16, 46%; npmSAH: *n* = 19, 54%). Demographic data are depicted in Table 1. All patients with npmSAH and pmSAH had a good grade on admission (Hunt and Hess 1–3) without statistical differences between all groups (*p* = 0.230). Modified Fisher scale was lowest in pmSAH patients, but comparable between aSAH and npmSAH (*p* = 0.2727). Patients with npmSAH were significantly older than patients with pmSAH. Smoking as an important vascular risk factor was highest in aSAH (*p* = 0.0028) without differences between npmSAH and pmSAH (see Table 1 and suppl.). Absolute serum glucose and rate of hyperglycemia on admission (glucose > 140md/dl) was highest in the npmSAH group (156 [134–189] mg/dl, resp. 72% hyperglycemia), while no patient in pmSAH presented with hyperglycemia (see Table 2 and suppl.). Likewise, serum glucose/potassium ratio showed significant differences between all groups (*p* = 0.0001) with again highest values in the npmSAH group (41.5 [32–48.25]mg/dl). Renal function parameters were significantly elevated in the npmSAH group compared to aneurysmal SAH (creatinine, *p* = 0.0029; glomerular filtration rate, *p* = 0.044) (see Table 2 and suppl.).

**Table 1:** Baseline demographics, imaging features, and past medical history. *aSAH* aneurysmal subarachnoid hemorrhage, *npmSAH* non-perimesencephalic SAH, *pm* perimesencephalic SAH; *p*-value, ANOVA/Kruskal–Wallis test/chi-square as appropriate (see Supplement material for multiple comparisons)

Parameter	aSAH	npmSAH	pmSAH	*p*-value
Total [*n*]	115	19	16	
Age [yrs]	55 [48–65]	60 [56–68]	52 [42–60]	**0.032**
Female sex [*n*]	75 (65.2%)	9 (47.4%)	6 (37.5%)	0.0512
Hunt and Hess [grade]				0.230
I	33 (28.6%)	6 (31.6%)	9 (56.3%)	
II	43 (37.4%)	8 (42.1%)	7 (43.7%)	
III	39 (33.9%)	5 (26.3%)	0 (0%)	
Modified Fisher grade	2 [1–3]	1 [1, 2]	1 [1]	**0.0005**
Loss of consciousness [n]	38/115 (33%)	0 (0%)	0 (0%)	**0.0004**
Past medical history and risk factors				
Antiplatelet treatment [n]	18 (15.7%)	3 (15.8%)	2 (10.5%)	0.946
Anticoagulation treatment (n)	5 (4.4%)	2 (10.5%)	0 (12.5%)	0.32
Arterial hypertension [n]	55 (47.8%)	12 (63.2%)	7 (50%)	0.415
Smoking	49 (42.6%)	3 (15.8%)	1 (6.25%)	**0.0028**

**Table 2:** Serum biomarkers on admission (patients only included when admission ≦ after ictus). *aSAH* aneurysmal subarachnoid hemorrhage, *npmSAH* non-perimesencephalic SAH, *pm* perimesencephalic SAH; ANOVA/Kruskal–Wallis test/chi-square as appropriate. For multiple comparisons, please see the supplementary material

Parameter	aSAH	npmSAH	pmSAH	*p*-value
Total [*n*] (admission ≦24 h)	93 (80.9%)	18 (94.7%)	13 (81.3%)	0.33
White blood cell count [1000/µl]	13 [9.85–15.85]	10 [7.83–16.33]	11.3 [9.3–14.5]	0.229
Potassium [mmol/l]	4.0 [3.6–4.2]	3.9 [3.8–4.2]	3.9 [3.7–4.1]	0.83
Glucose/potassium ratio	34 [27–40]	41.5 [32–48.25]	28 [26–30]	**0.0003**
Glucose [mg/dl]	133.0 [110–157]	156 [134–189]	108 [95–120]	** < 0.0001**
Glucose > 140 mg/dl	39 (41.9%)	13 (72%)	0 (0%)	**0.0003**
Creatinine [mg/dl]	0.7 [0.6–0.8]	0.82 [0.74–1.0]	0.78 [0.63–0.96]	**0.0019**
Glomerular filtration rate [ml/min]	99.8 [87.7–107.3]	86.9 [71.3–100.4]	93.4 [84.2–109.2]	**0.048**
C-reactive protein [mg/l]	2.7 [1.25–4.5]	2.6 [0.7–3.13]	2.15 [1.5–5.6]	0.48
Lactate [mmol/l]	1.5 [1.0–2.3]	1.7 [1.4–2.8]	1.6 [0.8–1.9]	0.21

### Clinical and radiological course and outcomes for all SAH types

**Fig. 2:**
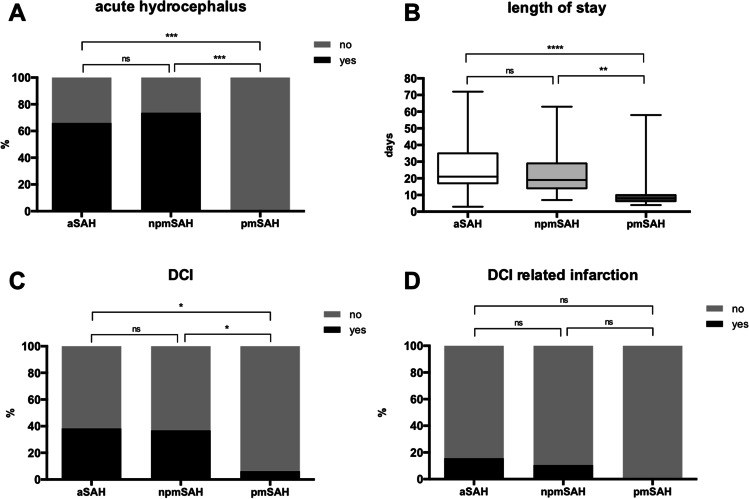
Clinical course and complications of the different SAH entities. **A** Rate of acute hydrocephalus (in percentage) was comparable between aSAH and npmSAH, but significantly lower in pmSAH. **B** Length of stay was longest in aSAH, but comparable to npmSAH (box-whisker, min–max). **C** Rate of DCI (in percentage) was comparable between aSAH and npmSAH, but lowest in pmSAH. **D** DCI-related infarction (in percentage) showed no differences between groups though missing in pmSAH. aSAH, aneurysmal subarachnoid hemorrhage (*n* = 115); npmSAH, non-perimesencephalic SAH (*n* = 19); pmSAH, perimesencephalic SAH (*n* = 16); DCI, delayed cerebral ischemia

**Fig. 3:**
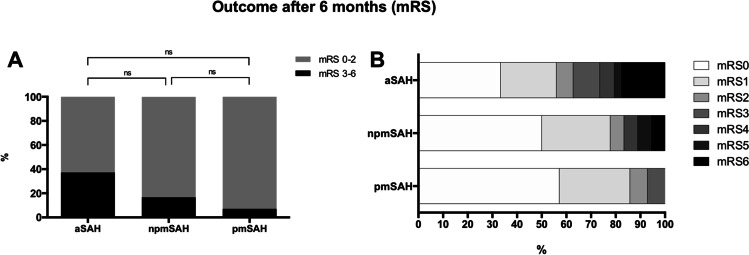
Neurological outcome after 6 months. **A** Dichotomized neurological outcome after 6 months is depicted for aSAH (*n* = 102), npmSAH (*n* = 18), and pmSAH (*n* = 14) (good = modified Rankin scale (mRS) 0–2, poor = mRS 3–6). Absolute numbers of poor outcome are highest in aSAH and lowest in pmSAH, but differences are not statistically significant in multiple comparison test. **B** All mRS grades for all groups without dichotomization. aSAH, aneurysmal subarachnoid hemorrhage; npmSAH, non-perimesencephalic SAH; pm, perimesencephalic SAH

Follow-up assessment after 6 months was completed in the majority of patients in all groups (see Table 3). Favorable outcome was reported in 13 of 14 pmSAH patients (92.9%) and 15 of 18 npmSAH patients (83.3%), whereas in the aSAH group, only 64 of 102 (62.7%) achieved a favorable outcome (*p* = 0.0264). However, multiple comparison showed no differences between groups (*p* = 0.11) (see Fig. 3 and suppl.). Patients with pmSAH had the shortest hospital stay (8 [6–10] days, *p* < 0.0001), while it was comparable for patients with aSAH and npmSAH (21 [17–35] days vs. 19 [14–29] days, *p* = 0.691) (see Fig. 2 and suppl.). Rate of acute hydrocephalus and need for permanent CSF diversion were also comparable for aSAH and npmSAH patients (66.1% vs. 73.7%, OR 0.696, 95% CI 0.234–2.074, *p* = 0.61, resp. 26.3% vs. 18.3%, OR 0.626, 95% CI 0.21–1.93, *p* = 0.53). In contrast, no acute hydrocephalus occurred in the pmSAH group (aSAH vs. pmSAH OR 63.91, 95% CI 3.7–1094, *p* < 0.0001; npmSAH vs. pmSAH OR 76.0, 95% CI 4.417–1774, *p* < 0.0001) (Fig. 2 and suppl.). The rate of DCI events was comparable in the npmSAH and aSAH group (38.5% vs. 36.8%, OR 1.06, 95% CI 0.389–2.9, *p* = 1.0), while there was only one DCI event in the pmSAH group (aSAH vs. pmSAH OR 9.3, 95% CI 1.19–72.9, *p* = 0.011, npmSAH vs. pmSAH OR 8.75, 95% CI 0.941–81, *p* = 0.047 resp.) (see Fig. 2 and suppl.). Accordingly, DCI-related infarction rate was also comparable between aSAH and npmSAH patients (15.7% vs. 10.5%, OR 1.6, 95% CI 0.33–7.42, *p* = 0.74) (see Fig. 2). Overall infarction rate (any etiology) was highest in patients with aSAH (*n* = 27, 46.6%) without statistical difference to npmSAH (*n* = 4, 23.5%) (OR 2.8, 95% CI 0.824–9.72, *p* = 0.103), while cerebral infarction was absent in patients with pmSAH. In-house mortality was also absent only in the pmSAH group but failed to reach statistical significance (aSAH vs. npmSAH OR 2.5, 95% CI 0.69–20.18, *p* = 0.69, pmSAH vs. npmSAH, OR 2.7 95% CI 0.1–70.4, *p* = 1.0) (see Table 3).

**Table 3:** Clinical and radiological outcomes. *aSAH* aneurysmal subarachnoid hemorrhage, *npmSAH* non-perimesencephalic SAH, *pm* perimesencephalic SAH, *DCI* delayed cerebral ischemia, *CSF* cerebrospinal fluid, *VP* ventriculoperitoneal, *mRS* modified Rankin Scale. ANOVA/Kruskal–Wallis test/chi-square as appropriate. For multiple comparisons, please see the supplementary material

Parameter	aSAH	npm SAH	pmSAH	*p*-value
DCI [*n*]	44 (38.3%)	7 (36.8%)	1 (6.25%)	**0.041**
DCI-related infarction [*n*]	18 (15.7%)	2 (10.5%)	0 (0%)	0.2095
Any infarction [*n*]	48 (43.6%)	4 (21.1%)	0 (0%)	**0.0011**
Acute hydrocephalus [*n*]	76 (66.1%)	14 (73.7%)	0 (0%)	** < 0.0001**
Need for permanent CSF diversion (VP shunt) [*n*]	21 (18.3%)	5 (26.3%)	0 (0%)	0.106
Length of stay [d]	21 [17–35]	19 [14–29]	8 [6–10]	** < 0.0001**
In-house mortality [*n*]	14 (12.2%)	1 (5.3%)	0 (0%)	0.173
mRS 0–2 at 6mo [*n*]	64/102 (62.7%)	15/18 (83.3%)	13/14 (92.9%)	**0.0264**

## Discussion

Non-aneurysmal subarachnoid hemorrhage is generally considered to follow a significantly more benign course with a better neurological outcome compared to aneurysmal SAH [2, 10]. Our data, however, highlights that patients with non-aneurysmal npmSAH carry a high risk for complications, such as DCI, DCI-related infarction, hydrocephalus, and poor neurological outcome, comparable to that of good-grade aneurysmal SAH (HH 1–3) patients. Patients with characteristic pmSAH, however, generally face a more benign clinical course, rarely with complications such as hydrocephalus, DCI, or DCI-related infarction. Such patients can be discharged earlier and usually have an excellent neurological outcome.

Previous studies on aneurysmal or non-aneurysmal SAH reported conflicting results regarding baseline characteristics and clinical course. A retrospective study on 154 non-aneurysmal SAH patients highlighted that elevated CRP and white blood cell count can predict a poor neurological outcome in patients with non-aneurysmal SAH [22]. In contrast, other retrospective studies suggested a significantly aggravated clinical course only to aneurysmal SAH [15, 17] but these cohorts consisted of aSAH of all clinical grades. A recent prospective study performed by Al Mufti et al. reported a lower risk for vasospasm but comparable risk for poor outcome and DCI for npmSAH compared to aSAH confirming our own results [1]. In our study, patients with pmSAH had a more benign clinical course. These results are in line with a recently published review by Mensing et al. who showed a minimal complication rate with no deaths and rebleeding events by analyzing 208 papers [16].

Our study comprises a prospective cohort of different SAH entities that was balanced for good neurological grade on admission. There are different explanations for our findings: first, npmSAH involves the complete basal cisterns and/or subarachnoid hemorrhage space and may thereby exert its detrimental effect in a similar fashion, compared to aSAH. Although there is no evident rupture of a radiologically detectable aneurysm, the pathological cascades leading to DCI and poor neurological outcome seem to be at least partly similar. The comparable subarachnoid blood load between aSAH and npmSAH (mod Fisher scale) in our cohort may support this theory. For pmSAH, conversely, where the subarachnoid blood load is lower and more localized, numerous smaller studies suggest various venous abnormalities as bleeding origin (for review see [11] and [16]). However, interpretation of the current literature is impeded because detailed differentiation of the entities pmSAH and npmSAH has not been performed. Given the important role of early brain injury for the development of complications subsequent to aSAH, it seems reasonable to assume different pathogeneses between all three entities, and furthermore, that pmSAH and npmSAH are separate entities of diverging severity. In an experimental setting, we recently showed that the bleeding velocity itself (as assumed to be lower in the presence of a venous bleeding source) has an impact on disturbed cerebral autoregulation and pronounced early neuronal cell loss [5]. However, the bleeding source in npmSAH and pmSAH remains unknown to date, and therefore, the true impact of the bleeding event is cryptic. Second, neurological outcome assessed by the mRS was predominantly favorable in all groups, but only in pmSAH all patients achieved excellent outcome, while 37% of good-grade aSAH and 17% of npmSAH patients had an unfavorable outcome, underlining the importance of close clinical observation in those groups (see Fig. 3).

Third, the dysregulation of glucose metabolism in the npmSAH group could also be an indicator for a more severe clinical course as already described for aSAH [4, 8, 9]: higher serum glucose variability and hyperglycemia on admission—possibly as a consequence of the stress reaction triggered by the hemorrhage—are associated with poor outcome [14]. These variables are also associated with clinical severity and thus the extent of stress hormone release and complication rate after aSAH [7, 18]. Data on glucose dynamics after non-aneurysmal SAH, however, are sparse. In our study, patients with npmSAH showed significantly higher overall glucose levels and more often presented with hyperglycemia on admission, thus possibly indicating a more severe clinical course. As npmSAH patients tended to be somewhat older in our cohort, a higher prevalence of previously undetected diabetes mellitus respectively impaired glucose tolerance may partially explain these findings. Also, hyperglycemia and higher variability in the above-mentioned studies on aSAH were primarily associated with disease severity, which in turn is a major determinant for outcome. Therefore, glucose levels may be less affected in cohorts limited to only good-grade SAH patients like ours. However, the interpretation of these findings is difficult and remains speculative in absence of serum cortisol profiles [26].

The strength of our study is the prospective design and an adequate sample of good-grade aSAH patients. However, there are also distinct limitations: the comparatively small number of patients in the pmSAH and npmSAH group is a limitation to our analysis, possibly owing to the prospective nature of our study and the comparatively long timeframe needed for recruitment. Additionally, the study lacks statistical power to provide absolute risk ratios and precluded a more rigorous matching process. Therefore, the study is not powered to detect a clinically meaningful difference in DCI rates between aSAH, pmSAH, and npmSAH. Especially the low number of patients with pmSAH (*n* = 16) precludes generalization of observed DCI rates, deaths, or treatment for hydrocephalus in this patient population. Our results should therefore be considered as rather hypothesis-generating and remain to be validated in a greater patient population. Additionally, we have not performed MRI scans in the acute phase in every SAH patient which would allow us a more detailed analysis of the subarachnoid blood clot distribution in relation to neuroanatomical structures between all entities. Neurological outcome assessment was performed after 6 months, which is a comparatively short time period for SAH, and the chosen outcome scale (mRS) is designed for daily routine performance and may miss more complex cognitive impairments.

In summary, our study is the first prospective analysis to relate both non-aneurysmal, non-perimesencephalic, and perimesencephalic bleeding patterns, with a balanced cohort of good-grade aSAH patients. Whether npmSAH and its aggravated clinical course is truly a separate entity with a different underlying pathophysiology remains to be determined.

We hypothesize that a more specific classification of these diverging entities is mandatory, while a pooling of these patients into a supposedly more benign group (“non-aneurysmal”) should be avoided. Frequent complications such as DCI and DCI-related infarction in patients with npmSAH once more emphasize the importance of close observation not only of patients with ruptured aneurysms, but also of patients with non-aneurysmal, but also non-perimesencephalic bleeding pattern.

As a consequence, we have adopted these findings into our daily practice and have extended our standard operating procedure for aSAH towards patients with npmSAH. Patients with respective bleeding patterns remain on our ICU on average at least 14 days in order to enable timely detection of complications, while transfer to the regular ward and early discharge is encouraged in pmSAH patients.

## Conclusion

In a prospective cohort of good-grade SAH patients, npmSAH and aSAH featured a similar though aggravated clinical course, advocating for prolonged neurological observation. Complications were largely absent in patients with classic pmSAH implying fundamental differences in pathophysiology and underscoring the need for differentiation of terminology and treatment in non-aneurysmal hemorrhage.

## Supplementary Information

Below is the link to the electronic supplementary material.

Supplementary file1 (DOCX 30 KB)

## Data Availability

The raw data of this analysis can be made available by the authors to any qualified researcher.

## Abbreviations

CT: Computed tomography
DCI: Delayed cerebral ischemia
DSA: Digital subtraction angiography
EVD: External ventricular drainage
GCS: Glasgow coma scale
HH: Hunt and Hess grade
ICP: Intracranial pressure
ICH: Intracerebral hemorrhage
MRI: Magnetic resonance imaging
ModFish: Modified Fisher scale
mRS: Modified Rankin scale
p_ti_O_2_: Partial pressure of brain tissue oxygen
aSAH: Aneurysmal subarachnoid hemorrhage
naSAH: Non-aneurysmal subarachnoid hemorrhage
npmSAH: Non-perimesencephalic subarachnoid hemorrhage
pmSAH: Perimesencephalic subarachnoid hemorrhage
aSDH: Acute subdural hematoma
VP shunt: Ventriculoperitoneal shunt
