# Supplementary material for: Baseline characteristics and outcome for aneurysmal versus non-aneurysmal subarachnoid hemorrhage: a prospective cohort study
Source: Neurosurg Rev. 2021 Oct 4;45(2):1413–20. doi: 10.1007/s10143-021-01650-x (PMC8976787; doi:10.1007/s10143-021-01650-x)
Supplement: Supplementary file 1 — Supplementary file1 (DOCX 30 KB) [file 10143_2021_1650_MOESM1_ESM.docx]

Supplementary material

Neurosurgical Research

**Baseline characteristics and outcome for aneurysmal versus non-aneurysmal subarachnoid hemorrhage: a prospective cohort study**

Catharina Conzen^1^, Miriam Weiss^1^, Walid Albanna^1^, Katharina Seyfried^1^, Tobias P. Schmidt^1^, Omid Nikoubashman^2^, Christian Stoppe^3^, Hans Clusmann^1^, Gerrit A. Schubert^1^

^1^ Department of Neurosurgery, University Hospital Aachen, RWTH Aachen University, Aachen, Germany

^2^ Department of Neuroradiology, University Hospital Aachen, RWTH Aachen University Aachen, Germany.

^3^ Department of Intensive Care and Intermediate Care, University Hospital Aachen, RWTH Aachen University, Aachen, Germany

**Corresponding author:**

Catharina Conzen, MD

Department of Neurosurgery, RWTH Aachen University Hospital

Pauwelsstrasse 30

52074 Aachen, Germany

Tel. no.: +49-241-8088481

E-mail address: cconzen@ukaachen.de

Table 1

| **parameter** |  | **aSAH** | **npmSAH** | **pmSAH** | **p-value** | |  |
| --- | --- | --- | --- | --- | --- | --- | --- |
| total, n |  | 115 | 19 | 16 |  | |  |
| median age [1q.-3.q] [yrs] |  | 55[48-65] | 60[56-68] | 52[42-60] | **0.0317** | |  |
|  |  |  | Mean Diff. | 95% CI of diff | Adjusted P Value | |  |
|  |  | npmSAH vs. aSAH | 10.77 | -13.7-1.36 | 0.138 | |  |
|  |  | pmSAH vs. aSAH | 1.544 | --2.6-13.1 | 0.2587 | |  |
|  |  | pmSAH vs. npmSAH | -12.32 | -1.13-21.09 | **0.025** | |  |
| Mod Fisher |  | 2[1-3] | 1[1-2] | 1[1-1] | **0.0005** | |  |
|  |  |  | Mean Diff. |  | Adjusted P Value | |  |
|  |  | npmSAH vs. aSAH | 16.79 |  | 0.2727 | |  |
|  |  | pmSAH vs. aSAH | 39.78 |  | **0.0006** | |  |
|  |  | pmSAH vs. npmSAH | 22.99 |  | **0.2735** | |  |
| Arterial hypertension |  | 55 (47.8%) | 12 (63.2%) | 7(43.7%) | 0.4154 | |  |
| Diabetes mellitus |  | 4 (3.5%) | 2 (10.5%) | 0 | 0.2398 | |  |
| Smoking habit |  | 49(42.6%) | 3(15.8%) | 1(6.25%) | **0.0028** | |  |
|  |  | npmSAH vs. aSAH |  |  | **0.0263** | |  |
|  |  | pmSAH vs. aSAH |  |  | **0.0196** | |  |
|  |  | pmSAH vs. npmSAH |  |  | 0.6768 | |  |
| **Serum biomarkers** | | | | | | | |
| total [n]  (admission ≦24h) |  | 41(70.7%) | 16(94.1%) | 12(85.7%) |  | |  |
| Glc/potassium ratio |  | 34[27-40] | 41.5[32-48.25] | 28[26-30] | **0.0003** | |  |
|  |  |  |  |  | Adjusted P Value | |  |
|  |  | aSAH vs. npmSAH |  |  | **0.035** | |  |
|  |  | aSAH vs. pmSAH |  |  | **0.018** | |  |
|  |  | npmSAH vs. pmSAH |  |  | **0.0002** | |  |
| glucose [mg/dl] |  | 133[111-157] | 156[134-189] | 108[95-120] | **0.0001** | |  |
|  |  |  | Mean rank diff, |  | Adjusted P Value | |  |
|  |  | aSAH vs. npmSAH | -18.73 |  | 0.0130 | |  |
|  |  | aSAH vs. pmSAH | 15.30 |  | 0.005 | |  |
|  |  | npmSAH vs. pmSAH | 34.89 |  | <0.0001 | |  |
| glucose > 140mg/dl |  | 14(34.2%) | 11(68.8%) | 0(0%) | **0.0008** | |  |
|  |  | aSAH vs. npmSAH |  |  | 0.0357 | |  |
|  |  | aSAH vs. pmSAH |  |  | 0.0231 | |  |
|  |  | npmSAH vs. pmSAH |  |  | 0.0003 | |  |
| Creatinine [mg/dl] |  | 0.7[0.6-0.8] | 0.82[0.74-1.0] | **0.78[0.63-0.96]** | **0.0019** | |  |
|  |  |  | Mean rank diff, |  | Adjusted P Value | |  |
|  |  | aSAH vs. npmSAH | -20.39 |  | **0.0029** | |  |
|  |  | aSAH vs. pmSAH | -10.31 |  | 0.248 | |  |
|  |  | npmSAH vs. pmSAH | 10.08 |  | >0.999 | |  |
| glomerular filtration rate [ml/min] |  | 99.8[87.7-107.3] | 86.9[71.3-100.4] | **93.4[84.2-109.2]** | **0.048** | |  |
|  |  |  | Mean Diff, |  | Adjusted P Value | |  |
|  |  | aSAH vs. npmSAH | 18.19 |  | **0.044** | |  |
|  |  | aSAH vs. pmSAH | 3.394 |  | 0.999 | |  |
|  |  | npmSAH vs. pmSAH | -14.79 |  | 0.25 | |  |
| **Clinical course** | | | | | |  |  |
| acute hydrocephalus, n (%) |  | 31(53.4%) | 13(76.5%) | 0 (0%) | **<0.0001** | |  |
|  |  | pmSAH vs. npmSAH |  |  | **<0.0001** | |  |
|  |  | pmSAH vs. aSAH |  |  | **0.0001** | |  |
|  |  | npmSAH vs. aSAH |  |  | 0.1029 | |  |
| DCI, n (%) |  | 20(34.5%) | 7(41.2%) | 0(0%) | **0.0234** | |  |
|  |  | pmSAH vs. npmSAH |  |  | **0.0087** | |  |
|  |  | pmSAH vs. aSAH |  |  | **0.0076** | |  |
|  |  | npmSAH vs. aSAH |  |  | 0.7746 | |  |
| Any infarction, n (%) |  | 27(46.6%) | 4(23.5%) | 0(0%) | **0.0025** | |  |
|  |  | pmSAH vs. npmSAH |  |  | 0.1075 | |  |
|  |  | pmSAH vs. aSAH |  |  | **0.0006** | |  |
|  |  | npmSAH vs. aSAH |  |  | 0.1029 | |  |
| VP shunt, n (%) |  | 6(10.3%) | 4(23.5%) | 0 (0%) | 0.11 | |  |
|  |  | aSAH vs. npmSAH |  |  | 0.23 | |  |
|  |  | aSAH vs. pmSAH |  |  | 0.59 | |  |
|  |  | \| npmSAH vs. pmSAH \| \| --- \| |  |  | 0.11 | |  |
| Length of stay |  | 21[17-35 | 19[14-29] | 8[6-10] | **<0.0001** | |  |
|  |  | aSAH vs. npmSAH |  |  | 0.6991 | |  |
|  |  | aSAH vs. pmSAH |  |  | <0.0001 | |  |
|  |  | \| npmSAH vs. pmSAH \| \| --- \| |  |  | 0.0028 | |  |
|  |  |  |  |  |  | |  |
| Outcome 6 months |  | 64/102 (62.7%) | 15/18 (83.3%) | 13/14 (92.9%) | **0.0264** | |  |
|  |  | aSAH vs. npmSAH | OR 2.96 | 95% CI  0.8064 to 10.93 | 0.11 | |  |
|  |  | aSAH vs. pmSAH | OR 7.7 | 95% CI 0.9704 to 61.40 | **0.0326** | |  |
|  |  | npmSAH vs. pmSAH | OR 2.6 | 95% CI 0.24 to 28.16 | 0.62 | |  |
| mRS 0 |  | 34 | 9 | 8 |  | | |
| mRS1 |  | 23 | 5 | 4 |  | | |
| mRS2 |  | 7 | 1 | 1 |  | | |
| mRS3 |  | 11 | 0 | 1 |  | | |
| mRS4 |  | 6 | 1 | 0 |  | | |
| mRS5 |  | 3 | 1 | 0 |  | | |
| mRS6 |  | 18 | 1 | 0 |  | | |
| Outcome 12 months |  | 63/95 (66.3%) | 6/8 (75%) | 7/7 (100%) | **0.0264** | | |
|  |  | aSAH vs. npmSAH | OR 1.5 | 95% CI  0.29 to 7.98 | 1.0 | | |
|  |  | aSAH vs. pmSAH | OR 7.7 | 95% CI 0.42 to 138.8 | 0.09 | | |
|  |  | npmSAH vs. pmSAH | OR 5.77 | 95% CI 0.23 to 143.5 | 0.467 | | |
